# A new recombinant avian metapneumovirus vaccine candidate provides complete protection against the novel variant infectious bursal disease virus in chickens

**DOI:** 10.1186/s13567-026-01735-9

**Published:** 2026-07-11

**Authors:** Lingzhai Meng, Yuntong Chen, Mengmeng Yu, Xiaole Qi, Yongzhen Liu, Suyan Wang, Xiaoxiao Xue, Tao Zhang, Wenrui Fan, Zibo Zhang, Ying Wang, Ru Guo, Mingxue Hu, Yanping Zhang, Yulu Duan, Hongyu Cui, Yulong Gao

**Affiliations:** 1 Avian Immunosuppressive Diseases Division, State Key Laboratory of Animal Disease Control and Prevention, Harbin Veterinary Research Institute, The Chinese Academy of Agricultural Sciences, Harbin, 150069 People’s Republic of China; 2 World Organization for Animal Health (WOAH) Reference Laboratory for Infectious Bursal Disease, Harbin Veterinary Research Institute, The Chinese Academy of Agricultural Sciences, Harbin, 150069 People’s Republic of China; 3 National Poultry Laboratory Animal Resource Center, Harbin, 150069 People’s Republic of China

**Keywords:** Novel variant infectious bursal disease virus, avian metapneumovirus subtype B, rLN16A-nVarVP2, VP2 gene, protective efficacy

## Abstract

Infectious bursal disease (IBD) is an acute, highly contagious, and immunosuppressive condition in chickens, caused by the infectious bursal disease virus (IBDV). The recent emergence of novel variant IBDV (nVarIBDV) poses a significant threat to the global poultry industry. However, currently available vaccines provide only limited protection against nVarIBDV strains. In this study, a recombinant avian metapneumovirus subtype B (aMPV/B) expressing the nVarIBDV VP2 gene (rLN16A-nVarVP2) was successfully rescued by inserting the gene between the G and L genes of the attenuated aMPV/B strain LN16-A. Immunofluorescence and Western blotting analyses confirmed stable VP2 expression in vitro. Further evaluation showed that VP2 insertion did not alter the growth kinetics of the parental virus, and the expression remained stable after 20 serial passages. A single immunization with rLN16A-nVarVP2 elicited robust humoral and cellular immune responses in specific pathogen-free chickens, inducing high levels of neutralizing antibodies against both nVarIBDV and aMPV/B, as well as Th1 (IL-2, IFN-γ) and Th2 (IL-4, IL-6) cytokines. Moreover, rLN16A-nVarVP2 conferred complete (100%) protective efficacy against both nVarIBDV and aMPV/B, fully prevented bursal atrophy and clinical signs associated with aMPV/B infection, and markedly decreased bursa viral load (nVarIBDV) and turbinate viral shedding (aMPV/B). Our findings suggest that rLN16A-nVarVP2 represents a promising live attenuated bivalent vaccine candidate for preventing infections caused by nVarIBDV and aMPV/B.

**Supplementary Information:**

The online version contains supplementary material available at 10.1186/s13567-026-01735-9.

## Introduction

Infectious bursal disease (IBD) is a highly contagious and immunosuppressive condition caused by the infectious bursal disease virus (IBDV), an economically important pathogen that affects the global poultry industry [1]. Several major pathogenic types of IBDV have been identified, including classical IBDV (cIBDV), variant IBDV (varIBDV) [2], very virulent IBDV (vvIBDV) [3], and novel variant IBDV (nVarIBDV) [4]. cIBDV was first isolated in the United States in 1957, followed by varIBDV in North America and Australia [5]. In the late 1980s, vvIBDV caused mortality rates exceeding 60% in Europe and subsequently spread across Asia, Africa, and South America [3]. Recently, vvIBDV has been gradually controlled through effective vaccination programs and improved feeding practices. Since 2017, nVarIBDV, genetically distinct from the earlier IBDV, has become widespread in vaccinated chicken farms in several countries, including China [4], Japan [6], Malaysia [7] and South Korea [8]. At present, nVarIBDV is spreading rapidly, even surpassing vvIBDV in prevalence, thereby posing new threats and challenges to the poultry industry [9, 10].

IBDV is a member of the genus *Avibirnavirus* within the family *Birnaviridae*, and its genome consists of two segments of double-stranded RNA [11]. Segment B encodes the VP1 protein, an RNA-dependent RNA polymerase that plays a key role in viral replication and evolution [12, 13]. Segment A encodes the VP2, VP3, VP4, and VP5 proteins, of which VP2 is the major structural protein of IBDV and the host-protective immunogen [14]. The neutralizing epitope of VP2 induces the production of neutralizing antibodies, thereby protecting the host against IBDV infection [15]. Consequently, various viral vector vaccines expressing the vvIBDV VP2 gene have been developed to control IBD, including those based on Marek’s disease virus (MDV) [16], Newcastle disease virus, and Fowl adenovirus (FAdV) [17].

Avian metapneumovirus (aMPV) is a member of the genus *Metapneumovirus* in the family Pneumoviridae and causes acute respiratory disease in turkeys and swollen head syndrome in chickens [18]. The aMPV genome is a nonsegmented, negative-stranded RNA of 13.2 kb containing eight genes arranged in the order of 3′-leader-N-P-M-F-M2-SH-G-L-trailer-5′ [19]. The International Committee of Virus Taxonomy has identified four subtypes of aMPV: aMPV/A, aMPV/B, aMPV/C, and aMPV/D [20]. Previous studies have shown that the metapneumovirus genome is highly flexible and can stably express foreign genes [21]. For example, insertion of the enhanced green fluorescent protein (EGFP) gene between the leader and N genes of the human metapneumovirus genome successfully enabled EGFP expression [22]. Additionally, Falchieri et al. inserted the S1 and N genes of infectious bronchitis virus (IBV) into the genome of aMPV/C to develop a bivalent recombinant vaccine that provided complete (100%) protective efficacy against both IBV and aMPV [23]. However, the potential of aMPV/B as a vaccine vector remains largely unclear.

Vaccination remains the most effective strategy for controlling IBD [24]. Traditional live attenuated and inactivated vaccines are crucial for controlling IBD, however, the limitations of these vaccines, including maternally derived antibodies (MDA) interference and the risk of virulence reversion, highlight the importance of novel vaccine platforms [25, 26]. The VP2 protein of IBDV has been the focus of subunit vaccine development due to its role as the major host-protective antigen. The subunit vaccines developed using *Escherichia coli* [27], yeast [28] and baculovirus [29] have been proved to produce high levels of neutralizing antibodies and protect against vvIBDV. Several virus vector vaccines developed by reverse genetics have also been demonstrated to prevent vvIBDV effectively. Zhang et al. constructed a recombinant FAdV serotype 4 (FAdV-4) bivalent inactivated vaccine expressing the vvIBDV VP2 protein, which conferred complete protection against both FAdV-4 and vvIBDV virulent strain [17]. In addition, Fan et al. constructed a recombinant MDV-vectored vaccine (MDV-VP2-HA), which confers simultaneous protection against avian influenza virus (AIV), vvIBDV, and MDV [30]. However, nearly all currently available commercial vaccines target vvIBDV and fail to confer adequate protection against nVarIBDV in vaccinated chickens [31, 32]. Therefore, antigen-matched vaccination is essential for the comprehensive prevention and control of nVarIBDV infection.

In previous study, we attenuated a virulent aMPV/B strain using serial passages in Vero cells and obtained an attenuated strain (LN16-A) [33]. Here, we first constructed a recombinant virus expressing the VP2 gene of nVarIBDV using the LN16-A as a vector. We then systematically evaluated the humoral and cellular immune responses induced by this recombinant virus, as well as its immunoprotective effects on nVarIBDV and aMPV/B.

## Materials and methods

### Cells, viruses, and plasmids

The virulent aMPV/B strain LN16-V (GenBank accession no. MH745147.1) and virulent nVarIBDV strain SHG19 (GenBank accession no. Segment A, MH879045; Segment B, MH879092) were isolated in our laboratory. Vero and BSR-T7/5 cells were cultured in Dulbecco's modified Eagle's medium supplemented with 10% fetal bovine serum at 37 °C in a humidified incubator with 5% CO_2_. The full-length plasmid pOK-LN16A containing attenuated aMPV/B strain LN16-A (GenBank accession no. PP069785) cDNA and four support plasmids (pCAGGS-N, pCAGGS-P, pCAGGS-M21, and pCAGGS-L) are all stored in our laboratory [34]. The expression plasmid pCAGGS-VP2 for expressing the VP2 gene of nVarIBDV is maintained in our laboratory [35]. A cell-adapted nVarIBDV strain, rGtSHG19VP2, was generated using reverse genetics [15].

### Construction of full-length cDNA of recombinant aMPV/B expressing the nVarIBDV VP2 gene

**Figure 1:**
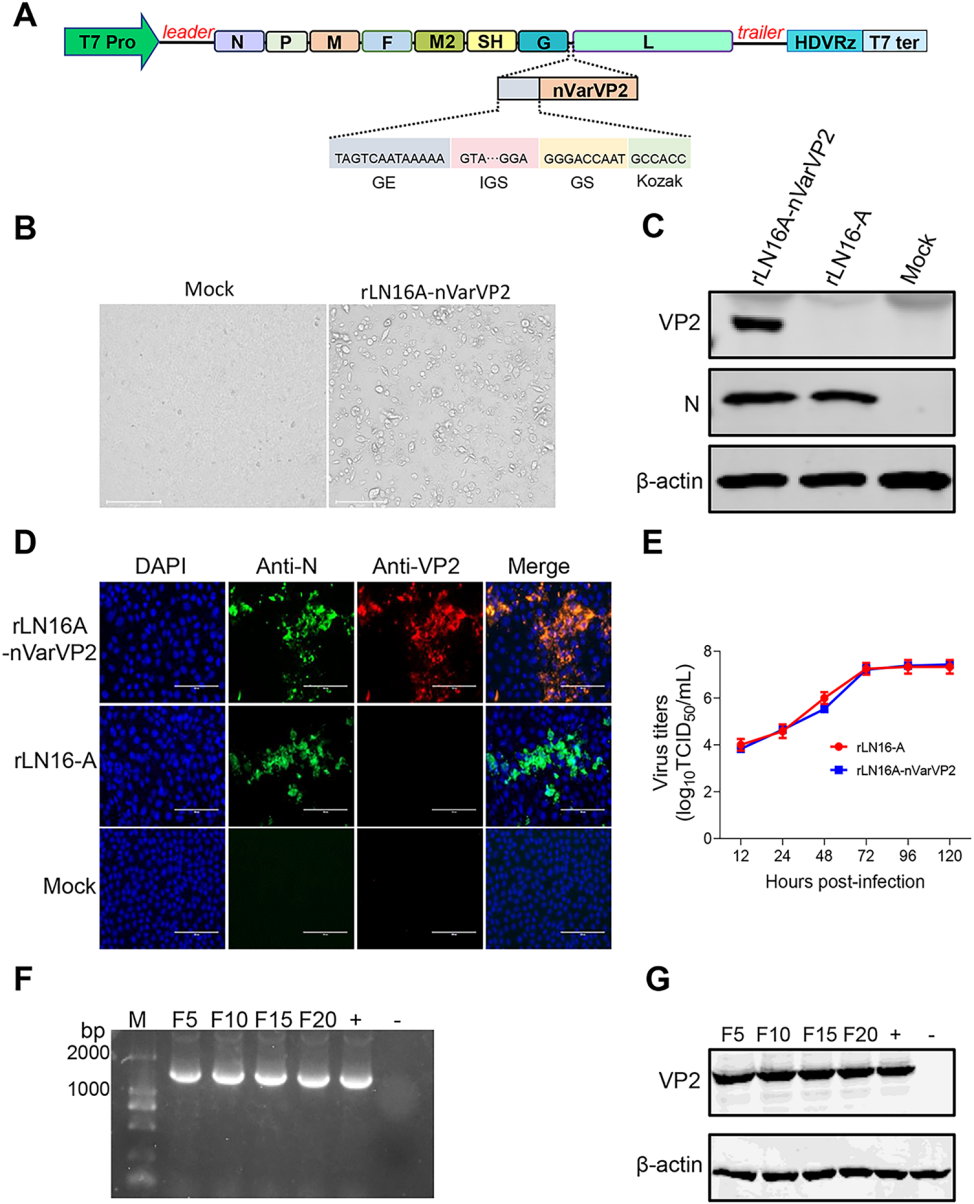
**Construction and rescue of recombinant avian metapneumovirus subtype B (aMPV/B) vaccine (rLN16A-nVarVP2) expressing the novel variant infectious bursal disease virus (nVarIBDV) VP2 gene**. **A** Schematic diagram of rLN16A-nVarVP2 construction. The gene end (GE) sequence of the G gene, the intergenic sequence (IGS) between G and L genes, the gene start (GS) sequence of the L gene, and the Kozak sequence were inserted upstream of the VP2 open reading frame to generate the VP2 expression cassette, which was subsequently cloned between the G and L genes. **B** Microscopic examination of rLN16A-nVarVP2-infected and uninfected Vero cells (mock). Scale bar: 275 μm. **C** Expression of VP2 protein in Vero cells infected with rLN16A-nVarVP2, detected by Western blotting. Vero cells infected with rLN16A-nVarVP2 or rLN16-A were subjected to Western blotting using a mouse anti-IBDV-VP2 monoclonal antibody and a rabbit anti-aMPV-N polyclonal antibody, with uninfected Vero cells used as a negative control. **D** Immunofluorescence analysis (IFA) of N (green) and VP2 (red) expression in rLN16A-nVarVP2. Vero cells infected with rLN16A-nVarVP2 or rLN16-A at a multiplicity of infection (MOI) of 0.01. The cells were detected with mouse anti-IBDV-VP2 monoclonal antibody and rabbit anti-aMPV-N polyclonal antibody, followed by a mixture of the FITC-labeled Goat Anti-Mouse IgG (H + L) (A0568, Beyotime, China) and Goat Anti-Rabbit IgG (H + L) (BN20636, Biorigin, China). Scale bar: 100 μm. **E** Comparison of replication kinetics between rLN16A-nVarVP2 and rLN16-A. Data are presented as the mean ± standard deviation (SD) from three independent experiments. **F** PCR amplification of the VP2 gene in different generations of rLN16A-nVarVP2. “ + ”: positive control. PCR amplification was performed using the pOKLN16A-nVarVP2 as template. “−”: negative control. PCR amplification was performed using reverse-transcribed cDNA from mock Vero cells as template. **G** Western blotting was performed on Vero cells infected with rLN16A-nVarVP2 from F5, F10, F15 and F20 generations using IBDV-VP2 monoclonal antibodies. The positive control was Vero cells transfected with the pCAGGS-VP2, with untreated Vero cells serving as the negative control.

Linearized vectors were generated after enzymatic cleavage of the pOK-LN16A plasmid with SpeI. Total RNA was extracted from bursae infected with the nVarIBDV SHG19 strain [36] using an EZ-10 Spin Column Total RNA Isolation Kit (B610583-0100; Sangon Biotech). Reverse transcription was performed to obtain cDNA, using a reaction volume of 20 μL, with TransScript® One-Step gDNA Removal and cDNA Synthesis Supermix (AT311-02; TransGen Biotech) protocol. According to the manufacturer instruction of PrimeSTAR® Max DNA Polymerase (R045Q; Takara, Japan), the cDNA was then polymerase chain reaction (PCR)-amplified using primers VP2-F and VP2-R (Table 1) to generate the VP2 expression cassette. The PCR procedure consisted of 35 cycles of 94 °C for 30 s, 55 °C for 30 s and 72 °C for 1 min. The reaction system and component volumes were prepared exactly as recommended by the manufacturer. The full-length recombinant plasmid, pOKLN16A-nVarVP2, expressing the nVarIBDV VP2 gene, was constructed by cloning the VP2 expression cassette into the linearized pOK-LN16A vector (Figure 1A). Viral rescue was performed by transfecting cells with pOKLN16A-nVarVP2 and four support plasmids, as described previously [34]. To verify successful viral rescue, Western blotting and immunofluorescence analysis (IFA) were performed using aMPV/B anti-N polyclonal antibodies [34] and IBDV anti-VP2 monoclonal antibodies [30]. The recombinant virus successfully rescued was designated rLN16A-nVarVP2.

**Table 1:** **Sequences of primers used for construction of VP2 expression cassette and for detection**

Name	Primer sequence (5′-3′)
VP2-F	GGACCAATGCCACCATGACAAACCTGCA
VP2-R	TTTTTATTGACTATTACCTTAGGGCCCGAATT
GL-F	AGCCCAACTGCAATACAGAAAAAC
GL-R	GGTCGACCTATAATGCAAGACC

### Virus growth curve

Vero cells were infected with the recombinant virus and parental virus (rLN16-A) at a multiplicity of infection (MOI) of 0.01 and incubated at 37 °C with 5% CO_2_, respectively. The experiment was performed with three independent replicates. For each replicate, cell samples were collected at 12, 24, 48, 72, 96, and 120 h post-infection and freeze-thawed once. The resulting harvested supernatants, containing extracellular and intracellular viral released by freeze–thaw lysis, were serially diluted tenfold (10^–1^–10^–6^) and used to infect Vero cells in 96-well plates, with six replicates per dilution. Viral titers were determined using the Reed–Muench method and expressed as the 50% tissue culture infective dose (TCID_50_)/mL.

### Genetic stability analyses

To evaluate the genetic stability of the recombinant virus, serial passages were performed in Vero cells for 20 generations. Total RNA was extracted from passages F5, F10, F15, and F20 and then reverse-transcribed into cDNA using the PrimeScript Strand cDNA Synthesis Kit (6210A; Takara, Japan). According to the manufacturer instruction of PrimeSTAR® Max DNA Polymerase (R045Q; Takara, Japan), PCR amplification of the inserted VP2 gene was performed using GL-F and GL-R primers (Table 1) with following procedure: 35 cycles of 94 °C for 30 s, 55 °C for 30 s and 72 °C for 1 min. The reaction system and component volumes were prepared exactly as recommended by the manufacturer. In addition, Western blotting was performed using IBDV-VP2 monoclonal antibody on Vero cells infected with F5, F10, F15, and F20 generation viral, as well as Vero cells transfected with pCAGGS-VP2 (positive control) and untreated Vero cells (negative control) [30].

### Evaluation of the immunogenicity and protective efficacy of recombinant aMPV/B in chickens

**Figure 2:**
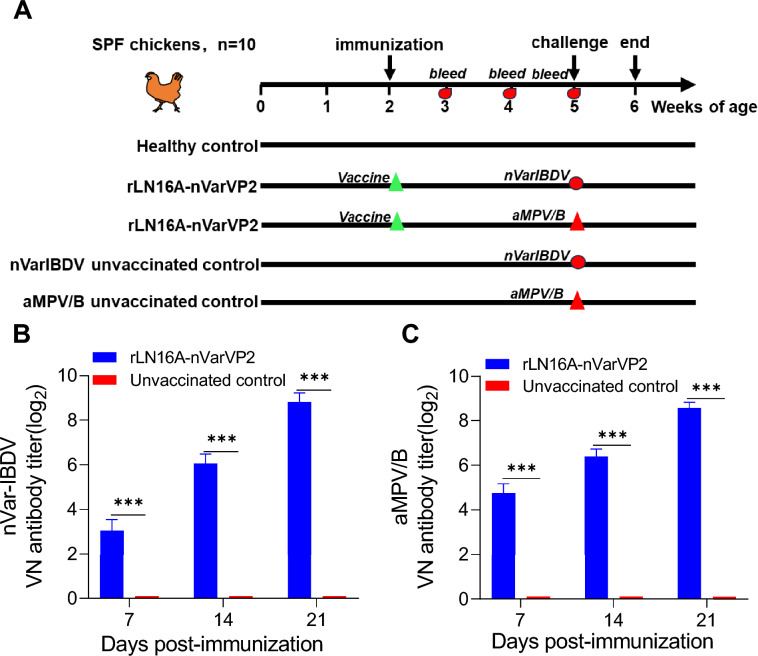
**Humoral immune responses in chickens vaccinated with rLN16A-nVarVP2**. **A** Schematic diagram of the animal experiment. One group (*n* = 10) served as a healthy control without treatment. Twenty specific pathogen-free (SPF) chickens were vaccinated with 200 μL of rLN16A-nVarVP2 via nasal inoculation, designated as the rLN16A-nVarVP2 vaccinated group. Another twenty chickens were vaccinated with 200 μL of phosphate buffered saline (PBS), designated as the unvaccinated group. At 21 days post-vaccination (dpv), 10 chickens from the rLN16A-nVarVP2 vaccinated group and 10 chickens from the unvaccinated control group were challenged with the nVarIBDV strain SHG19. For the remaining 10 chickens in each group, 200 μL of virulent aMPV/B strain LN16-V was administered. **B** Neutralizing antibodies titers against nVarIBDV measured following rLN16A-nVarVP2 vaccination. **C** Neutralizing antibodies titers against aMPV/B measured after rLN16A-nVarVP2 vaccination. Data are presented as the mean ± SD (*n* = 10). ***p < 0.001.

Fifty specific pathogen-free (SPF) 21-day-old White Leghorn chickens were randomly allocated into three groups to evaluate the immunogenicity and protective efficacy of the recombinant virus against the virulent aMPV/B strain LN16-V and nVarIBDV strain SHG19. The chickens were kept in negative-pressure-filtered air isolators and received feed and water ad libitum. Twenty chickens were vaccinated intranasally with 200 μL of recombinant virus (5000 TCID_50_ chicken^−1^), designated as the vaccinated group. Another twenty chickens received 200 μL phosphate buffered saline (PBS), designated as the unvaccinated control group. The remaining 10 unvaccinated chickens served as healthy controls (Figure 2A). Blood samples were collected from vaccinated and unvaccinated groups at 7, 14, and 21 days post-vaccination (dpv). Serum levels of cytokines, including IL-2 (ECH0041; Fine Test, China), IL-4 (SEKCN-0008; Solarbio, China), IL-6 (JL21628-48 T; JONLNBIO, China), and IFN-β (SEKP-0046; Solarbio, China), were quantified according to the manufacturer’s enzyme-linked immunosorbent assay (ELISA) instructions. At 21 dpv, 10 chickens from the vaccinated group and 10 from the unvaccinated control group were housed separately to prevent cross-contamination. Subsequently, these chickens were challenged with 10 BID_50_^−1^ (ten 50% birds infection dose per chicken) of the nVarIBDV SHG19 strain via intranasal and ocular administration. Chickens were euthanized and examined for bursal lesions at 7 days post-challenge (dpc). Body and bursa weights were recorded to calculate the bursa-to-body weight index (BBIX = bursal weight/body weight × 1000). The remaining 10 chickens from each of the vaccinated and unvaccinated control groups were challenged with 200 μL of the virulent aMPV/B strain LN16-V (5000 TCID_50_) via nasal inoculation. These chickens were also housed independently in separate isolators post-challenge to prevent cross-contamination. Clinical symptoms were monitored daily from 1–7 dpc, and the morbidity was calculated [33]. In addition, choanal swabs from chickens challenged with the LN16-V strain were collected to measure aMPV/B viral shedding at 1–7 dpc, as previously described [37]. Chickens were euthanized and examined at 7 dpc, with the turbinate fixed in 10% formalin for subsequent histopathological analysis.

### Virus neutralization assay

The serum samples were collected from vaccinated and unvaccinated groups at 7, 14, and 21 dpv. Collected serum samples were incubated at 56 °C for 30 min to inactivate complement. To measure neutralizing antibodies levels induced by rLN16A-nVarVP2, the serum was serially twofold diluted and mixed with 200 TCID_50_ of the rGtSHG19VP2 strain at 37 °C for 1 h. The serum–virus mixtures were then inoculated with DF-1 cells for 72 h, after which cytopathic effects (CPE) were observed. In addition, the serum was serially twofold diluted and mixed with 200 TCID_50_ of the LN16-V strain at 37 °C for 1 h. The serum–virus mixtures were then inoculated with Vero cells for 7 days to determine the levels of neutralizing antibodies against aMPV/B, as previously described [33].

### Reverse transcription quantitative polymerase chain reaction (RT-qPCR)

At 7 dpc, total RNA was extracted from bursa following challenge with SHG19 of nVarIBDV using RNAiso Plus reagent (9108Q, TaKaRa, Japan). According to the instructions, total RNA was reverse-transcribed into cDNA using the BioRT Master HiSensi cDNA First Stand Synthesis kit (BSB40T1, Bioer, China). To quantify the viral load of nVarIBDV in the bursa, RT-qPCR was performed using the target gene (VP5) forward primer VP5-F (5′-GAGCCTTCTGATGCCAACAAC-3′), the reverse primer VP5-R (5′-CAAATTGTAGGTCGAGGTCTCTGA-3′), and the IBDV probe (5′FAM-CGGCGTCCATTCCGGACGAC-3′BHQ). The housekeeping gene forward primer 28 s-F (5′-GGCGAAGCCAGAGGAAACT-3′), the reverse primer 28 s-R (5′-GACGACCGATTTGCACGTC-3′), and the 28 s probe (5′FAM-AGGACCGCTACGGACCTCCACCA-3′TAMRA). As previously described [17, 38], nVarIBDV viral load was calculated as the number of viral copies per 10^6^ cells (LoD: 30 copies/μL). To quantify the level of aMPV/B viral shedding, total RNAs were extracted from choanal swabs of chickens challenged with LN16-V at 1–7 dpc. Subsequently, RNA was reverse-transcribed into cDNA using the HiScript II Q RT SuperMix for qPCR. Based on the established standard curve [37], aMPV/B viral shedding was quantified by RT-qPCR (target gene: G) and expressed as the number of genome copies per milliliter (LoD: 80 copies/μL).

### Histopathological analysis

Bursae and turbinate tissues were fixed in 10% formalin and embedded in paraffin. Sections were stained with hematoxylin and eosin and examined for pathological changes.

### Statistical analyses

The normality of experimental data was first verified using the Shapiro–Wilk test, which indicated that the data did not conform to a normal distribution. Therefore, non-parametric statistical methods were employed for comparisons. Data were analyzed using one-way ANOVA through Prism 8 (GraphPad Software Inc., San Diego, CA, USA). Results are expressed as the mean ± standard deviation. Statistical significance was defined as *p < 0.05, **p < 0.01, and ***p < 0.001.

## Results

### Construction and recovery of recombinant aMPV/B expressing the nVarIBDV VP2 gene

The recombinant aMPV/B expressing the nVarIBDV VP2 gene was constructed by co-transfecting the full-length plasmid pOKLN16A-nVarVP2 and four support plasmids (pCAGGS-N, pCAGGS-P, pCAGGS-M21, and pCAGGS-L) into BSR/T7-5 cells. After the second passage, visible CPE, including clustering, detachment, and syncytial plaques, was observed (Figure 1B). No CPE was observed in negative control group, confirming the successful rescue of the recombinant virus, designated as rLN16A-nVarVP2. Western blotting analysis of rLN16A-nVarVP2-infected cells showed positive reactivity with aMPV-N polyclonal antibodies and IBDV-VP2 monoclonal antibodies, revealing an N-specific band at approximately 43.4 kDa and a VP2-specific band at approximately 48.5 kDa (Figure 1C). IFA detected N expression using aMPV-N polyclonal antibodies and VP2 expression with IBDV-VP2 monoclonal antibodies (Figure 1D). Moreover, the growth kinetics results showed that rLN16A-nVarVP2 replicated in Vero cells with efficiency comparable to that of the parental virus rLN16-A, demonstrating that the insertion of the VP2 gene did not affect viral replication (Figure 1E). Next, we evaluated the genetic stability of rLN16A-nVarVP2 across 20 rounds of in vitro serial passaging. PCR amplification and sequencing (not shown) confirmed the expected sequence of VP2 gene size (1886 bp) in various generations of the recombinant virus (Figure 1F). In addition, Western blotting further confirmed that the VP2 gene remained expression across various generations of the recombinant virus, indicating high genetic stability (Figure 1G).

### Humoral immune responses induced by rLN16A-nVarVP2

To evaluate the humoral immune responses induced by rLN16A-nVarVP2, 14-day-old SPF chickens were vaccinated with 5000 TCID_50_ per bird. As shown in Figure 2B, the average neutralizing antibodies titer against nVarIBDV in the rLN16A-nVarVP2 vaccinated group was 3.1 log_2_ at 7 dpv and gradually increased to 6 log_2_ at 14 dpv and 8.8 log_2_ at 21 dpv. The average neutralizing antibodies titers against aMPV/B in rLN16A-nVarVP2 vaccinated group were 4.8, 6.4, and 8.6 log_2_ at 7, 14, and 21 dpv, respectively (Figure 2C). In contrast, chickens in the unvaccinated group did not produce neutralizing antibodies against either nVarIBDV or aMPV/B. These results suggest that a single immunization with rLN16A-nVarVP2 induces neutralizing antibodies responses against both nVarIBDV and aMPV/B in SPF chickens.

### Cell-mediated immune responses induced by the rLN16A-nVarVP2

**Figure 3:**
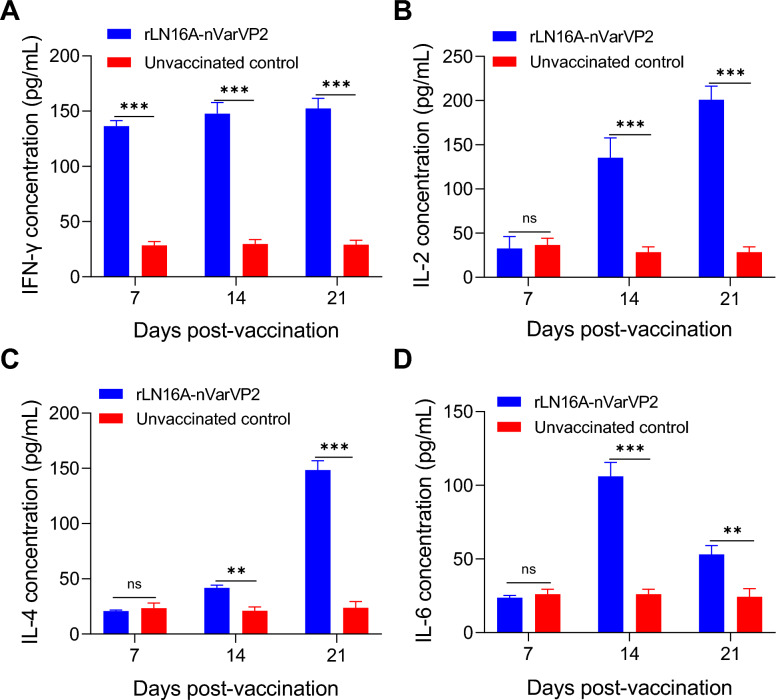
**Cell-mediated immune responses in chickens vaccinated with rLN16A-nVarVP2**. (**A** and **B**) Serum levels of IFN-γ (**A**) and IL-2 (**B**) in rLN16A-nVarVP2 vaccinated chickens. **C** and **D** Serum levels of IL-4 (**C**) and IL-6 (**D**) in rLN16A-nVarVP2 vaccinated chickens. T helper (Th) 1 (Th1; IL-2 and IFN-γ) and Th2 (IL-4 and IL-6) cytokine concentrations were measured via ELISA. Data are presented as the mean ± SD (*n* = 10). **p < 0.01; ***p < 0.001.

To evaluate cell-mediated immune responses induced by rLN16A-nVarVP2, serum concentrations of Th1 (IFN-γ and IL-2) and Th2 (IL-4 and IL-6) cytokines were quantified using ELISA. At 7 dpv, the average IFN-γ concentration in rLN16A-nVarVP2 vaccinated group (136 pg/mL) was approximately 4.8-fold higher than that in the unvaccinated control group (Figure 3A), while the levels of the other three cytokines (IL-2, IL-4 and IL-6) were comparable to those in unvaccinated control group (Figures 3B, C and D). At 14 and 21 dpv, the rLN16A-nVarVP2 vaccine group exhibited gradually increasing levels of IFN-γ, IL-2, and IL-4, which were significantly higher than those in unvaccinated control group. Peak concentrations reached 201 pg/mL, 152 pg/mL, and 148 pg/mL, respectively. Furthermore, the IL-6 levels in rLN16A-nVarVP2 vaccinated group gradually decreased, remaining significantly higher than those in unvaccinated control group at 14 and 21 dpv. These results suggest that a single immunization with rLN16A-nVarVP2 effectively stimulates both Th1 and Th2 cytokines.

### Protective efficacy of rLN16A-nVarVP2 against nVarIBDV and aMPV/B in chickens

**Figure 4:**
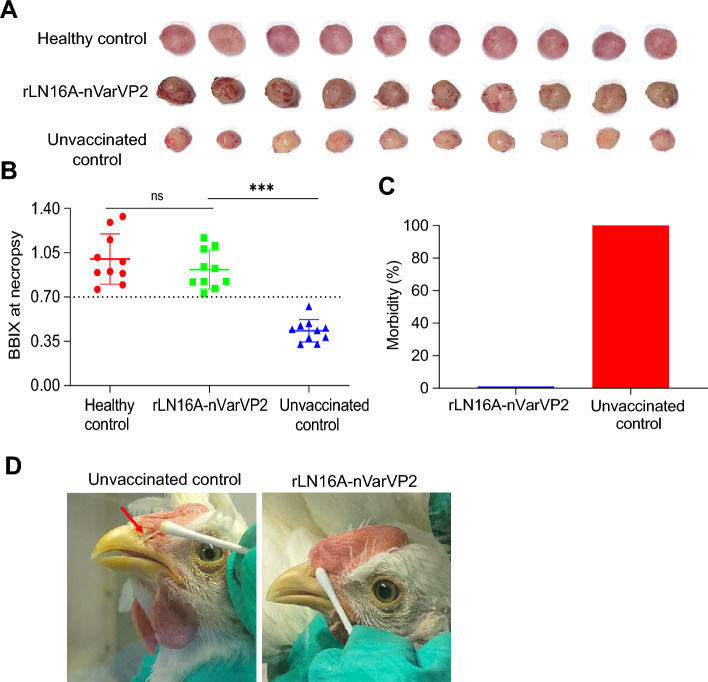
**Protective efficacy of rLN16A-nVarVP2 against nVarIBDV and aMPV/B**. **A** Gross morphology of the bursa after challenge with the nVarIBDV SHG19 strain. None of the bursae in the rLN16A-nVarVP2 vaccinated or healthy control groups showed atrophy, whereas those in the unvaccinated control group exhibited significant atrophy, yellowing, and inflammatory exudates. **B** Bursa-to-body-weight index (BBIX) in chickens (*n* = 10) following challenge with SHG19. A BBIX less than 0.7 was indicative of bursal atrophy. Data are presented as the mean ± SD (*n* = 10). ***p < 0.001. **C** Morbidity in chickens (*n* = 10) following challenge with virulent aMPV/B strain LN16-V. Morbidity was assessed based on the presence of nasal scabs or turbid nasal fluid. **D** Nasal fluid examination in chickens after challenge with LN16-V.

To evaluate the protective efficacy of rLN16A-nVarVP2 against nVarIBDV, 10 chickens from the rLN16A-nVarVP2 vaccinated group were challenged with nVarIBDV strain SHG19 at 21 dpv, while 10 chickens from the unvaccinated group served as challenge controls. Compared with the healthy controls, all bursae from the unvaccinated group showed significant atrophy, yellowing, and inflammatory exudates (Figure 4A) with BBIX of less than 0.7. In contrast, no lesions were observed in the bursae of the rLN16A-nVarVP2 vaccinated group, and the BBIX remained above 0.7 (Figure 4B). Furthermore, 10 chickens from rLN16A-nVarVP2 vaccinated and 10 from the unvaccinated group were challenged with the virulent aMPV/B strain LN16-V at 21 dpv. Unvaccinated chickens developed cloudy, viscous nasal fluid at 3 dpc, reaching 100% morbidity by 6 dpc (Figure 4C). In contrast, all rLN16A-nVarVP2 vaccinated chickens were completely protected and exhibited no clinical signs of disease (Figure 4D). These results suggest that rLN16A-nVarVP2 provides complete (100%) protection against challenge with nVarIBDV and aMPV/B in chickens.

### Determination of nVarIBDV viral loads and aMPV/B viral shedding

**Figure 5:**
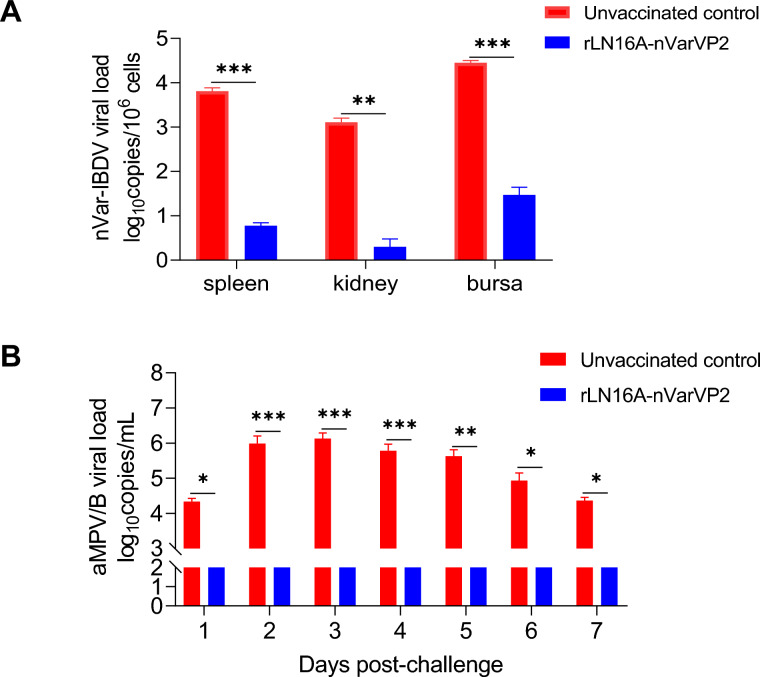
**Determination of nVarIBDV viral load and aMPV/B viral shedding**. **A** Viral load of nVarIBDV in the spleens, kidneys, and bursae (*n* = 3 each) from rLN16A-nVarVP2 vaccinated and unvaccinated control groups. **B** Viral shedding of aMPV/B in rLN16A-nVarVP2 vaccinated and unvaccinated control groups challenged with LN16-V. Data are presented as the mean ± SD (*n* = 10). *p < 0.05; **p < 0.01; ***p < 0.001.

To further evaluate the efficacy of rLN16A-nVarVP2 as a vaccine candidate, nVarIBDV viral loads in bursae and aMPV/B viral shedding in choanal swabs were quantified. Following challenge with the nVarIBDV strain SHG19, the average viral loads in the bursae, kidneys, and spleens of chickens in the unvaccinated control group were 10^4.5^, 10^3.1^ and 10^3.5^ copies/10^6^ cells, respectively, at 7 dpc (Figure 5A). In contrast, the average viral loads in the bursae, kidneys, and spleens of chickens in the rLN16A-nVarVP2 vaccinated group were reduced by 10^3.3^, 10^2.7^ and 10^3.2^ -fold, respectively, after challenge with SHG19, at 7 dpc. Furthermore, after challenge with the aMPV/B virulent strain LN16-V, unvaccinated chickens began shedding aMPV/B at 2 dpc, reaching a peak of 10^6.1^ copies/mL at 3 dpc. However, almost no aMPV/B shedding was detected in choanal cleft swabs of chickens from the rLN16A-nVarVP2 vaccinated group, with a maximum value of only 10^2.4^ copies/mL—10^3.7^-fold lower than in the unvaccinated chickens (Figure 5B). These results demonstrate that rLN16A-nVarVP2 effectively reduces nVarIBDV viral load in the bursa and markedly decreases aMPV/B shedding in the upper respiratory tract.

### Histopathological analysis of bursa and turbinate tissues

**Figure 6:**
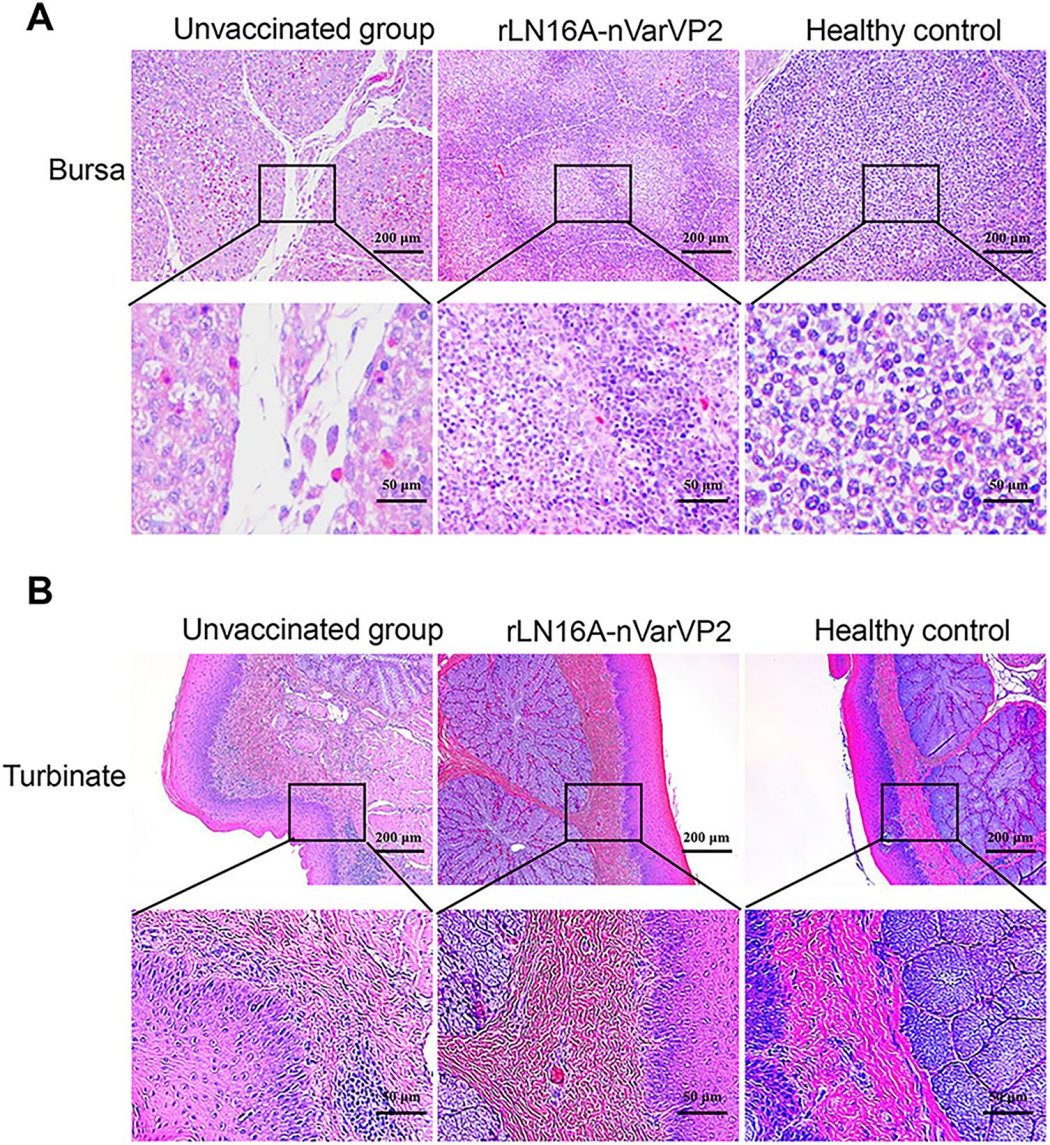
**Histopathological analysis of bursa and turbinate tissues.** **A** Representative images showing histopathological changes in bursal tissue from unvaccinated, vaccinated, and healthy control chickens (*n* = 3 per group). **B** Representative images showing histopathological changes in turbinate tissue from unvaccinated control, rLN16A-nVarVP2 vaccinated, and healthy control chickens (*n* = 3 per group). Scale bars: 200 μm.

Histopathological examinations were performed on bursae and turbinate tissues from chickens challenged with the nVarIBDV strain SHG19 and aMPV/B virulent strain LN16-V at 7 dpc. Following challenge with SHG19, bursae in the unvaccinated control group showed severe pathological damage, including follicular atrophy, mesenchymal hyperplasia, and marked lymphocyte depletion (Figure 6A). In contrast, no pathological damage was observed in the bursae of chickens in both the rLN16A-nVarVP2 vaccinated and healthy control groups. In addition, multiple inflammatory cell infiltrations were observed in the turbinate submucosa of the unvaccinated group following challenge with LN16-V, whereas no pathological lesions were observed in the turbinate tissues of the vaccinated or healthy control groups (Figure 6B). These results suggest that rLN16A-nVarVP2 effectively prevents the development of target-organ lesions following challenge with nVarIBDV and aMPV/B.

## Discussion

The continuous evolution of IBDV, particularly the emergence of vvIBDV and nVarIBDV, posed significant challenges to the prevention and control of IBD [39, 40]. Recent epidemiological findings in China have shown that the nVarIBDV has emerged as the dominant epidemic strain in chicken flocks [41]. Although nVarIBDV does not cause the same high levels of mortality as vvIBDV, it can significantly damage the bursa, resulting in atrophy, and cause severe long-term immunosuppression [4, 42]. In addition, current live attenuated, inactivated, and subunit vaccines against vvIBDV do not provide complete protection against nVarIBDV, leading to an annual increase in disease incidence [43]. Therefore, the development of a novel and more effective vaccine to control nVarIBDV infections is essential. As a metapneumovirus, the aMPV genome encodes only a few viral proteins but can accommodate one or two foreign genes, which is advantageous for stimulating specific immune responses and expressing foreign antigens [23, 44]. In addition, since aMPV primarily infects the respiratory tract of avians, vaccination with an aMPV vector induces not only long-lasting humoral but also cellular immunity. In our previous study, we developed an attenuated LN16-A strain through serial passaging in Vero cells. Furthermore, during five passages of LN16-A in chickens, no mutations were observed in the key genes F and G and no morbidity or pathological lesions were found in any of chickens, which showed no risk of reversion to virulence in vivo [33]. In this study, we successfully rescued a recombinant aMPV/B expressing the nVarIBDV VP2 gene (rLN16A-nVarVP2) using LN16-A as a vector. VP2 remained stably expressed even after 20 serial passages in vitro, indicating that rLN16A-nVarVP2 is highly stable. More importantly, rLN16A-nVarVP2 provided complete protection against both viral challenges, highlighting its potential as a bivalent vaccine.

Neutralizing antibodies levels are an important indicator for evaluating vaccine immunization efficacy [45]. Owing to the high immunogenicity of the IBDV VP2 protein, it has been widely used for purification and expression in vitro. Wang et al. constructed a recombinant *Lactococcus* expressing the VP2 gene of nVarIBDV, which induced moderate neutralizing antibodies levels (2^4^–2⁶) in chickens after immunization [46]. In addition, a recombinant FAdV-4 expressing the vvIBDV VP2 gene was inactivated and subsequently used to immunize SPF chickens, inducing neutralizing antibodies levels of approximately 2^8^ at 21 dpv [17]. In this study, a single immunization with rLN16A-nVarVP2 induced higher neutralizing antibodies levels (2^8.6^) than the two recombinant vaccines, suggesting that the novel aMPV/B vector can deliver foreign genes more efficiently.

In addition to the humoral immune response, T cells also play a crucial role in eliminating IBDV particles [47]. CD4^+^ helper T (Th) cells can be classified into Th1 and Th2 subsets according to the type of cytokines they produce [48]. Th1 cells secrete cytokines such as IL-2 and IFN-γ, which have been shown to enhance the clearance of intracellular pathogens [49]. In this study, serum concentrations of Th1 cytokines in the rLN16A-nVarVP2 vaccinated group were significantly higher than those in the unvaccinated control group, even at 21 dpv. Additionally, Th2 cells stimulate B cells to produce IL-6, which enhances the neutralization of pathogens by antibodies [50]. As a crucial pro-inflammatory cytokine, IL-6 plays dual roles in the regulation of immune responses. During the acute phase (7–14 dpv) after rLN16A-nVarVP2 vaccination, IL-6 acts as a pro-inflammatory mediator, activating innate immunity and promoting B-cell differentiation. When the immune response transitions to the adaptive memory phase (14–21 dpv), B cells have fully differentiated and matured, with antibodies titers increasing significantly, thus leading to a decreased demand for IL-6. In contrast, the levels of other cytokines, including IFN-γ, IL-2 and IL-4, either remain stable or increased, indicating a well-regulated immune resolution process. Therefore, the IL-6 level in the vaccinated group decreased at 21 dpv. This phenomenon is consistent with the findings of our previous studies [33]. Therefore, the elevated levels of Th1 and Th2 cytokines induced by rLN16A-nVarVP2 likely play a critical role in clearing nVarIBDV, which may explain the significant reduction in bursal viral load observed in vaccinated chickens. These findings further suggest that, in addition to increased neutralizing antibodies levels, cell-mediated immunity contributes to protection against nVarIBDV infection.

Vaccination remains the most effective strategy for preventing IBD [51]. However, due to differences in the antigenicity of commercial vaccines against nVarIBDV [15, 35], the available vaccines have been less effective in preventing pathological damage to the bursa caused by nVarIBDV infections. Hou et al. vaccinated 21-day-old SPF chickens with a commercial IBDV immune complex vaccine (W2512 strain) and observed that all chickens exhibited atrophied follicular bursa with waxy yellow jellies following challenge with the nVarIBDV FJ2019-01 strain at 21 dpv [43]. In addition, a combination of recombinant HVT vectors expressing the vvIBDV VP2 gene of the live attenuated B87 strain provided only 80% protective efficacy against nVarIBDV [52]. In this study, a single immunization with rLN16A-nVarVP2 completely prevented the development of bursal lesions and pathological damage, further demonstrating that the antigenic match of VP2 is critical for protection against nVarIBDV infection. The robust protection in SPF chickens indicates the promising field potential of rLN16A-nVarVP2. MDA interference poses a significant challenge for traditional IBDV vaccines, as live attenuated IBDV vaccines are unable to overcome high levels of MDA present in chicks [53]. Since rLN16A-nVarVP2 is not neutralized by IBDV-specific MDA, it can be administered in the presence of these antibodies, thereby enhancing its protective efficacy during the critical first few weeks of chicken life. Furthermore, consideration should be given to co-expressing the VP2 genes of nVarIBDV and other IBDV subtypes within the aMPV/B genome to develop broad-spectrum IBDV vaccines with enhanced antigenic matching. To the best of our knowledge, this represents the first systematic evaluation of the immunological efficacy of nVarIBDV VP2 using aMPV/B as the vector. These results will be valuable for the development of novel multivalent-vector avian vaccines.

In conclusion, we developed the first bivalent vaccine candidate expressing the nVarIBDV VP2 gene using the attenuated aMPV/B strain LN16-A as the backbone, which provides complete protection against both nVarIBDV and aMPV/B. Both in vitro and in vivo evaluations demonstrated that rLN16A-nVarVP2 is safe and effective, with important implications for the development of novel multivalent vector avian vaccines. Such bivalent vaccine candidates have significant potential for improving disease control and reducing economic losses in the global poultry industry.

## Supplementary Information

**Additional file 1.** **Western blotting and PCR.**

## Data Availability

All data are fully available without restriction.
