# Supplementary material for: A new recombinant avian metapneumovirus vaccine candidate provides complete protection against the novel variant infectious bursal disease virus in chickens
Source: Vet Res. 2026 Jul 11;57:132. doi: 10.1186/s13567-026-01735-9 (PMC13355354; doi:10.1186/s13567-026-01735-9)

Original figure

Fig. 1C

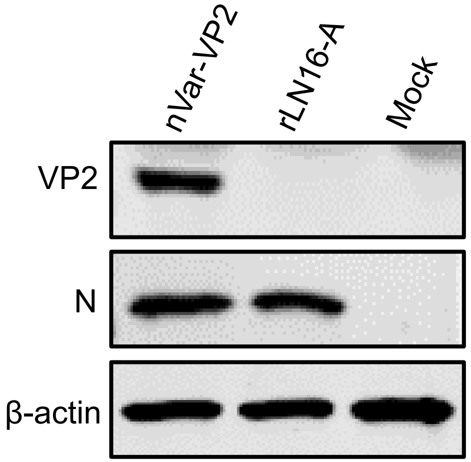

Fig. 1C-Original figure
(VP2 protein)

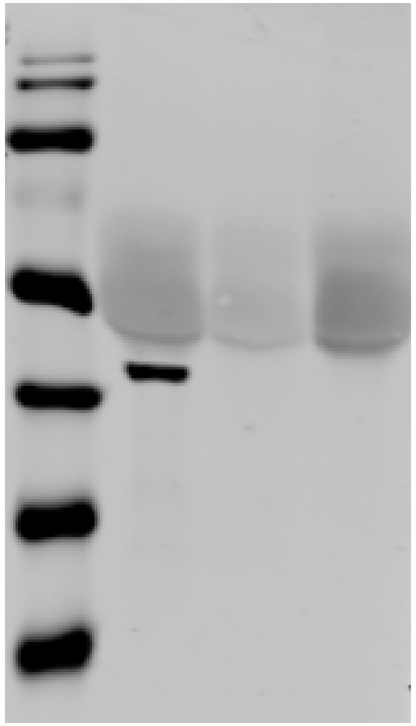

Fig. 1C-Original figure
(N protein)

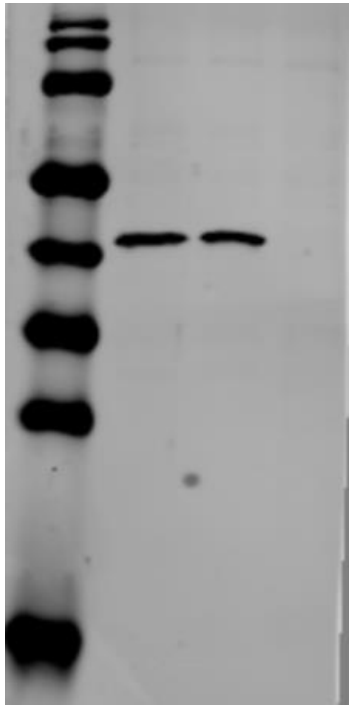

Fig. 1C-Original figure
(β-actin protein)

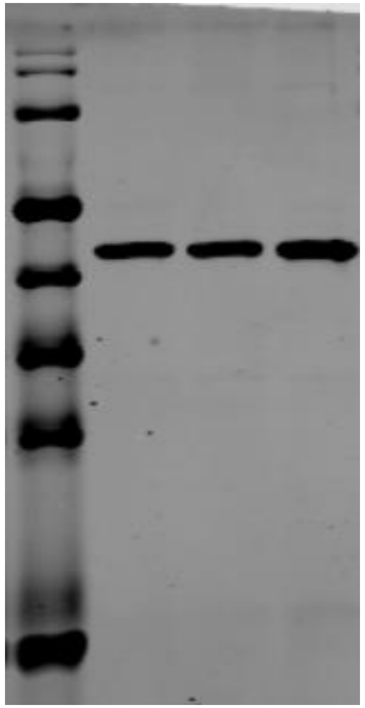

Original figure

Fig. 1F

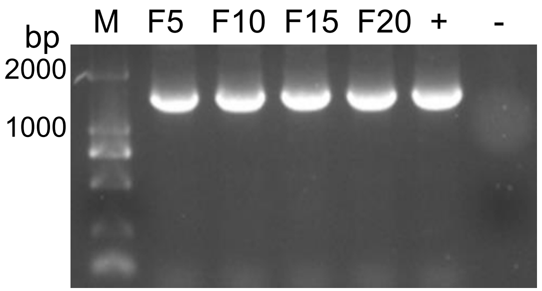

Fig. 1F-Original figure

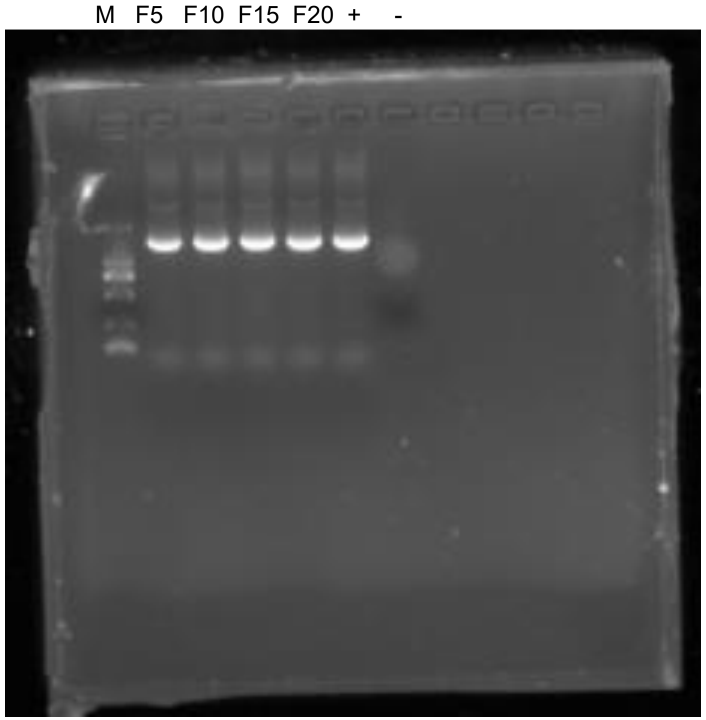

Original figure

Fig. 1G

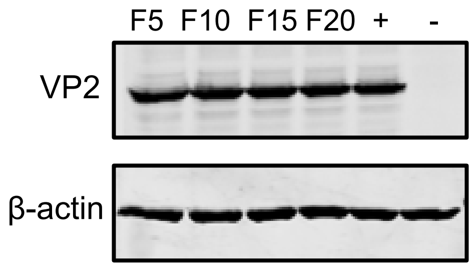

Fig. 1G-Original figure
(VP2 protein)

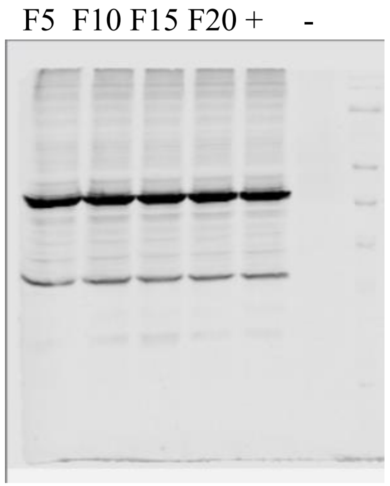

Fig. 1G-Original figure
(β-actin)

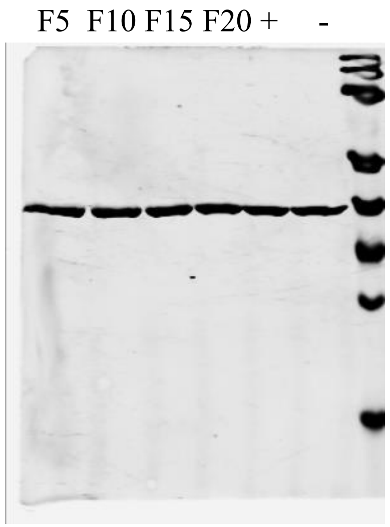

Supplement: Supplementary file 1 — Additional file 1. Western blotting and PCR. [file 13567_2026_1735_MOESM1_ESM.pdf]
